# How Redundant Are Redundant Color Adjectives? An Efficiency-Based Analysis of Color Overspecification

**DOI:** 10.3389/fpsyg.2016.00153

**Published:** 2016-02-19

**Authors:** Paula Rubio-Fernández

**Affiliations:** Centre for the Study of Mind in Nature, University of OsloOslo, Norway

**Keywords:** redundancy, color adjectives, object requests, informativeness, efficiency, pertinence, referential contrast

## Abstract

Color adjectives tend to be used redundantly in referential communication. I propose that redundant color adjectives (RCAs) are often intended to exploit a color contrast in the visual context and hence facilitate object identification, despite not being necessary to establish unique reference. Two language-production experiments investigated two types of factors that may affect the use of RCAs: factors related to the efficiency of color in the visual context and factors related to the semantic category of the noun. The results of Experiment 1 confirmed that people produce RCAs when color may facilitate object recognition; e.g., they do so more often in polychrome displays than in monochrome displays, and more often in English (pre-nominal position) than in Spanish (post-nominal position). RCAs are also used when color is a central property of the object category; e.g., people referred to the color of clothes more often than to the color of geometrical figures (Experiment 1), and they overspecified atypical colors more often than variable and stereotypical colors (Experiment 2). These results are relevant for pragmatic models of referential communication based on Gricean pragmatics and informativeness. An alternative analysis is proposed, which focuses on the efficiency and pertinence of color in a given referential situation.

## Introduction

Redundancy is generally defined in terms of informativeness: to say that an expression is redundant is to say that it is over-informative or overspecific (Engelhardt et al., 2006; Sedivy, 2007; Davies and Katsos, 2010; Arts et al., 2011a,b). According to this view the following utterances are redundant:

(a) ? John is a bachelor and he is unmarried.
(b) ? Today we are meeting at 7 pm in the evening.
(c) ? Give me the blue cup (uttered in a situation where there is only one cup).

While the first two examples are redundant because they are repetitive (e.g., a bachelor is unmarried by definition), the last example is redundant because it includes a non-contrastive use of a color adjective (i.e., ‘blue’ is not used to distinguish the intended cup from another cup of a different color). This paper focuses on the last type of redundant expressions; namely, redundant color adjectives (RCAs) in object requests. Unlike other types of speech acts involving reference, object requests require that the hearer visually identify the object in the physical environment as part of the pragmatic process of reference assignment. This feature of object requests makes them ideal for a pragmatic investigation of the role of visual processes in the production of referential expressions – which is the aim of the present study.

According to Grice’s (1975) Maxim of Quantity, speakers should try to provide their interlocutors with as much information as they need, but not more. Thus, in a situation where there is only one cup, the unmodified referential expression ‘the cup’ should be preferred to ‘the blue cup,’ other things being equal. Contrary to this theoretical expectation, experimental research has shown again and again that people tend to use adjectives redundantly in referential communication (e.g., Pechmann, 1989; Sedivy, 2003; Maes et al., 2004; Koolen et al., 2013). Another recurrent finding in the literature is that color adjectives tend to be used redundantly more often than other types of adjectives, especially relative adjectives such as ‘large’ or ‘small’ (Pechmann, 1989; Belke and Meyer, 2002; Nadig and Sedivy, 2002; Arts et al., 2011a,b).

The study reported in this paper investigated what factors affect the production of RCAs as a way to understand why they are so frequently used in object requests. Before I turn to that issue, I will address a theoretical question that has often been discussed in the pragmatics literature on referential communication: is color encoded because it is salient for the speaker or for the hearer? This question is important for the pragmatic analysis of color overspecification that I will propose next, which is based on efficiency.

### The Two Sides of Color Salience

As with other pragmatic aspects of reference production (e.g., articulatory attenuation; Brennan et al., 2010), it has often been discussed whether overspecification is a ‘speaker-internal’ or ‘hearer-oriented’ process (Arnold, 2008). Some authors have suggested that using adjectives redundantly may be easier for the speaker because it precludes the need to determine whether or not a certain adjective is necessary for unique reference (Pechmann, 1989; Belke and Meyer, 2002; Belke, 2006; Engelhardt et al., 2006; Koolen et al., 2013). It has also been argued that an overspecified description may help the hearer identify the intended object (Sonnenschein and Whitehurst, 1982; Mangold and Pobel, 1988; Nadig and Sedivy, 2002; Paraboni et al., 2007; van der Sluis and Krahmer, 2007; Arts et al., 2011a,b).

In the case of attenuation, it has been argued that attenuating the articulation of a word that is predictable in the context (vs. a word encoding new information) may be easier for the speaker insofar as it requires less articulatory effort than pronouncing it clearly (as is often done with new information). It is therefore possible that articulatory attenuation is simply easier for the speaker and benefits the hearer’s comprehension only fortuitously (but cf. Galati and Brennan, 2010). The case of overspecification is somewhat different, however, since identifying a property of a referent and encoding it in an utterance is generally harder (or more costly) for the speaker than not doing so. Since overspecification happens precisely in contexts where the encoded property is not necessary to establish unique reference, speakers’ choice of a longer referential expression needs to be explained.

One way in which overspecification may be easier for the speaker is by eliminating the search for potential competitors (i.e., objects of the same category as the intended referent) in the visual display. Pechmann (1989) observed that speakers often started producing overspecific referential expressions before they had finished scanning a display, suggesting redundancy may indeed facilitate reference production for the speaker. It must be noted, however, that this kind of evidence only explains the use of redundant adjectives in relatively large displays where scanning would be time consuming, but not in sparse displays where the speaker could determine at a glance that all objects are different.

However, even when overspecification would save the speaker the time to scan a display for potential competitors, such behavior would not be only ‘for the speaker’, but also ‘for the hearer.’ Thus, if a speaker’s referential strategy is to use a modified expression to preempt a possible ambiguity in a large display, then that default strategy is in itself evidence of audience design (while also being economical for the speaker). By contrast, a truly ‘egocentric’ speaker who was insensitive to the hearer’s perspective would not bother scanning the display or specifying a property of the intended referent in case there was a competitor: a self-centered speaker who made an object request would simply produce a bare definite description and leave it up to the hearer to ask for more information or make a guess (in the event that the request turned out to be underspecific).

I want to argue further that, at least in face-to-face communication, trying to decide whether overspecification is for the speaker or for the hearer is pretty much futile. Given that in face-to-face communication the physical environment is part of the common ground between the speaker and the hearer (Clark and Marshall, 1981), what is salient for the speaker (e.g., the color of a cup) will generally be salient for the hearer as well. Most importantly, a Gricean speaker is entitled to assume that much when producing a referential expression. In other words, since speakers and hearers rely on the same perceptual mechanisms, a cooperative speaker is entitled to assume that anything that is perceptually salient to him will also be salient for his interlocutor when they share a physical environment.

Experimental pragmatics studies have repeatedly found that the interlocutors’ sharing of a physical environment (what is known as ‘co-presence’) affects referential communication. For example, speakers’ eye gaze can be used by hearers to assign reference to a linguistic expression in face-to-face communication (e.g., Richardson and Dale, 2005; Hanna and Brennan, 2007; Neider et al., 2010). Likewise, bearing in mind the goal of the task at hand can also help hearers disambiguate referring expressions in interactive games (e.g., Chambers et al., 2002, 2004; Hanna and Tanenhaus, 2004). Co-presence can also affect language production, as when speakers tell stories to interlocutors who either share a picture of the story with the speaker, or rely entirely on the speaker’s narrative. In the latter condition, speakers tend to specify atypical objects more often than when these objects are visible to both interlocutors (Lockridge and Brennan, 2002). In this study I will argue that co-presence is relevant for the use of RCAs in object requests insofar as RCAs may facilitate object identification for the hearer.

In the remainder of this paper I will not try to discern whether speakers use RCAs in object requests because (a) they themselves find the color of the referent salient, or because (b) their interlocutor must find the color of the referent salient. Since interlocutors in face-to-face communication can normally assume that if (a) then (b), the speaker’s and hearer’s perspectives do not differ enough to be disentangled experimentally in such situations (for discussion, see Keysar, 1997; Brennan et al., 2010). Instead, I will treat reference as a ‘collaborative process’ between interlocutors (Clark and Wilkes-Gibbs, 1986) and try to argue that overspecification may be *efficient* in face-to-face communication.

In formulating an object request, the speaker’s goal is to get the hearer to identify the object in the physical environment and, assuming the hearer is willing to comply with the request, both interlocutors come to share the same goal. Object requests in face-to-face communication are therefore an ideal test case for the view that referential communication requires verbal and visual coordination between interlocutors, from which it follows that some referential expressions may be more efficient than others.

### Informativeness vs. Efficiency

Unlike computational psycholinguistics studies of reference production (e.g., Paraboni et al., 2007; Arts et al., 2011b; Koolen et al., 2013; Westerbeek et al., 2015), pragmatic accounts of referential communication have thus far failed to take into account perceptual factors in referential communication. For example, Sedivy (2003, 2004, 2007; Grodner and Sedivy, 2011) proposed a pragmatic analysis of color adjectives based on ‘default descriptions,’ according to which the default description of variable-color objects (e.g., a cup) includes a color adjective, while the default description of stereotypical-color objects (e.g., a banana) does not include a color adjective. This distinction explains why requests for variable-color objects tend to include RCAs, while requests for stereotypical-color objects only include color adjectives if there is a competitor in the display (e.g., a green banana; Sedivy, 2003, 2004; see also Westerbeek et al., 2015).

However, Sedivy’s account does not take perceptual factors into account, even though the physical environment is part of the common ground between interlocutors in face-to-face communication. According to Sedivy’s model, the referential expression ‘the blue cup,’ for example, would be redundant or ‘non-contrastive,’ if there is only one cup in the display. However, if we consider visual object identification as part of the pragmatic process of reference resolution in object requests, a pragmatic analysis of the expression ‘the blue cup’ must take into account not only the number of cups in the display, but also the colors of the other objects. Compare in this respect a visual search for a blue cup in a display where the cup is the only blue object, with the same visual search when all the objects in the display are blue. According to the standard pragmatic view, the referential expression ‘the blue cup’ would be equally over-informative in both contexts (so long as there was only one cup in each display). However, in the analysis I am proposing, the same referential expression would not be equally *inefficient*, since knowing the color of the cup would facilitate object identification in the polychrome display but not in the monochrome display.

Contrary to previous accounts, I want to propose that a pragmatic analysis of referential communication needs to be cast in terms of *efficiency* rather than *informativeness*. As was explained in the introduction, redundancy is traditionally described in terms of informativeness. However, such an analysis is only appropriate for statements, whose goal is to inform the hearer of a state of affairs (e.g., ‘It’s raining’); object requests, by contrast, are not informative as such. In terms of efficiency, a linguistic expression would be redundant if there was a more succinct alternative that would have achieved the goal of the speech act equally well. Given the goal of an object request, an optimal referential expression in an object request is one that allows the hearer to identify the intended object in the most efficient way. According to this view, RCAs should be understood as more or less efficient in a given context rather than being necessarily considered pragmatically infelicitous for being over-informative (Engelhardt et al., 2006, 2011).

An account of referential communication in terms of efficiency has the advantage that efficiency is a finer-grained notion than the standard three-way distinction between ‘underspecific,’ ‘minimal’ and ‘overspecific referential expressions,’ which has characterized Gricean analyses so far (e.g., Heller et al., 2012; Pogue et al., 2015). First of all, an efficiency-based analysis must take into account the specificity of a referential expression, since an underspecific referential expression (e.g., asking for ‘the cup’ in a situation where there are two cups) is less efficient than a minimal referential expression that establishes unique reference (e.g., ‘the blue cup’ in the same situation) insofar as the former expression leaves the hearer to choose randomly or ask for clarification.

In addition, looking at efficiency allows a deeper analysis of referential overspecification. For example, referring to the only cup in a display as ‘the blue cup’ would be more efficient if the cup was the only blue object than if there was also a blue jug in the display. However, color distinctiveness is not the only factor that may affect the relative efficiency of a referential expression: the number of objects in the display is also relevant. Thus, mentioning the color of the cup in a display of four objects would not be very efficient if two of them were blue, but the same expression would be considerably more efficient if the two blue objects were among 10 other objects of a different color. An analysis of referential communication based on efficiency is therefore much finer-grained than standard analyses in terms of informativeness. In this respect, while the idea that color may facilitate object identification is hardly new (e.g., Sonnenschein and Whitehurst, 1982; Mangold and Pobel, 1988; Paraboni et al., 2007; Arts et al., 2011a), the proposal to analyse RCAs as more or less efficient in a given context is novel and departs, in important ways, from standard pragmatic analyses in terms of informativeness.

### The Two Sides of Efficiency

An efficient referential expression is one that facilitates the identification of the intended referent for the hearer relative to other referential expressions. In the case of RCAs in object requests, a direct measure of efficiency would require comparing the speed of hearers’ identification of the referent following minimal and modified instructions (e.g., ‘Give me the cup’ vs. ‘Give me the blue cup’ in the same display containing only one cup). In a recent eye-tracking study investigating this particular question, I collected continuous eye-tracking measures of target identification and response times, and found an advantage for the modified instructions (containing RCAs) in all measures and across all conditions (which included different types of visual display; see Rubio-Fernández, under review). The results of this study therefore confirm that redundancy can be efficient, contrary to what standard pragmatic models have assumed to date.

Hearers’ eye movements and response times provide a direct measure of efficiency in referential communication when comparing modified and unmodified instructions. However, comparative comprehension data are not available to speakers and therefore do not inform their choice of referential expression. In that sense, comprehension data provide only half the picture – as one would expect. When speakers formulate an object request in face-to-face communication, what they have at their disposal is visual information about the environment in which their interlocutor must identify the target object (including the object’s contrastive properties). Therefore, reference production studies must also be carried out in order to establish whether speakers use RCAs when they can gauge that it may be efficient for their interlocutor in the visual context.

For example, a speaker who produces an efficient object request should be sensitive to the density of the display from which their interlocutor must select the referent. More specifically, such a speaker should have a stronger tendency to produce RCAs the denser the display is with objects, since that increases the difficulty of the hearer’s visual search. The tendency to provide our interlocutors with more information when they are looking for an object in a cluttered environment is generally an efficient strategy, which can be investigated in a language-production study without having to measure the speed of the interlocutor’s response (see Paraboni et al., 2007; Clarke et al., 2013; Rubio-Fernández, under review).

In line with the above arguments, the present study only investigated factors affecting the production of RCAs in relation to the potential efficiency of such uses in the given visual context. I was therefore not concerned with the actual effect that using (or not using) RCAs may have on reference resolution (since such differential effects do not inform a speaker’s choice of referential expression in the first place). In this sense, I will only consider efficiency from the viewpoint of the speaker: an efficient referential expression is one that the speaker *could reasonably expect* to help the hearer identify the intended referent in the visual context. This pragmatic notion of efficiency is broadly related to the speaker’s cooperative intention, and is not dependent on whether the referential expression is actually effective for the hearer.

### Factors Affecting the Use of Redundant Color Adjectives in Object Requests

It has been suggested before that factors other than considerations of unique reference may affect the choice of an adjective in an object request; for example, high-frequency adjectives and adjectives for salient properties are likely to be used in definite descriptions (Pechmann, 1989; Sedivy, 2004; Koolen et al., 2011). The present study investigated two types of factors that may affect the production of RCAs: visual-contextual factors and semantic-category factors.

Visual-contextual factors affect the use of RCAs in relation to the efficiency of color in a given situation; that is, to the extent that color may help the interlocutor identify the intended object. Two specific hypotheses were tested in relation to visual-contextual factors: first, RCAs are more efficient in an object request if the objects in the display are of different colors than if they are all the same color, especially if the referent is the only object of its color. This is so because color can be used to identify the intended referent in a polychrome display, but not in a monochrome one. I therefore predicted that more RCAs would be produced in polychrome displays than in monochrome displays. Such a difference was reported by Belke and Meyer (2002) and Koolen et al. (2013), although not in connection with the hearer’s visual search for the referent.

Second, since language processing is incremental, color adjectives are a more efficient cue to the hearer’s visual search in pre-nominal position than in post-nominal position. Eye-tracking studies have shown that a spoken instruction guides the hearer’s eye movements incrementally (Spivey et al., 2001; Reali et al., 2006; Clarke et al., 2015). Thus, when an English interlocutor processes the overspecific instruction ‘Give me the *blue* cup,’ she uses the color adjective to guide her visual search for the cup. In contrast, when a Spanish interlocutor processes the overspecific instruction ‘Dame la taza *azul*,’ she starts looking for the cup before she gets to process the color adjective, possibly finding the referent without using its color as a cue. Therefore, even if adjective position is a syntactic constraint, it affects the hearer’s visual search for the referent – hence its classification as a visual-contextual factor in this study. Given the difference in efficiency between pre-nominal and post-nominal RCAs, I predicted that more RCAs would be produced in English than in Spanish.

Semantic-category factors affect the use of color adjectives in relation to the noun that they modify, and hence according to our world knowledge of the category. For example, Sedivy (2003, 2004) found that color adjectives are used redundantly in requests for objects of variable colors (e.g., cups) but not of stereotypical colors (e.g., bananas). Two hypotheses were tested in relation to semantic-category factors: first, following up on Sedivy’s findings, the present study investigated the use of RCAs for objects of atypical colors (e.g., a pink banana). It was predicted that RCAs would be used more often for atypical- than for variable- and stereotypical-color objects, since atypical-color objects violate our expectations about a given category. This hypothesis was recently supported by the results of Westerbeek et al. (2015), who modified the color of fruits and vegetables (which normally have stereotypical colors) in a referential communication study.

Second, I propose that color is more important or pertinent for some semantic categories than for others (e.g., clothes and cars, on the one hand, vs. geometrical figures and tools, on the other) and predict that people will produce more RCAs when color is pertinent for a given semantic category. The pertinence of color for a given category should have an effect on the frequency with which color adjectives are used to refer to that category, as suggested by collocations with nouns for which color is a central property (e.g., ‘little black dress,’ ‘black tie,’ ‘white collar workers’ or ‘red sports car’). Underlying such frequency effects, however, it is possible that both speakers and hearers recognize an optimal level of description for any given category.

Just as referring to one’s pet as ‘my dog’ is normally more appropriate than as ‘my animal’ (Reiter, 1991; Geurts, 2010), it is not unlikely that specifying the color of a certain object is generally appropriate on the grounds that color is a central property for the category (e.g., ‘the black pen’ vs. ‘the black radio’). In such instances of color overspecification, Dale and Reiter (1995) have suggested that speakers may use ‘reference scripts’ that determine which properties are expected for a certain semantic category (for the related notion of ‘default descriptions,’ see Sedivy, 2003, 2004; Grodner and Sedivy, 2011). Thus, even if color might not necessarily be efficient in the visual context, it could be argued that specifying the color of clothes and shoes, for example, is generally pertinent for the requested object and therefore acceptable (and maybe even expected by the interlocutor, according to their reference script).

## Experiment 1

The first experiment in the study investigated two hypotheses related to the efficiency of RCAs in object requests. First, whether speakers would produce more RCAs in polychrome displays in which the referent was the only object of its color, than in monochrome displays where all objects were the same color as the target. This pattern of results was previously observed by Belke and Meyer (2002) and Koolen et al. (2013). However, these studies investigated other factors in addition to the number of colors in the display (e.g., the effect of size and orientation contrast in the visual display). It is therefore not possible to establish to what extent these results were due to the effect of color contrast. In fact, Koolen et al. (2013) argue that it is ‘scene variation’ (generally understood as the number of dimensions along which the objects of a display may vary) which drives the use of RCAs in their study, and not color contrast *per se*.

There is also a methodological reason why the results of Belke and Meyer (2002) and Koolen et al. (2013) may not be conclusive: in both studies monochrome and polychrome trials were interspersed and it is therefore possible that the RCAs that were observed in monochrome trials were a carry-over effect from previous polychrome trials. Belke and Meyer (2002), for example, observed that color adjectives were overspecified in monochrome displays of geometrical figures up to 66.5% of the time. However, it is an open question whether their participants would have produced such a high proportion of RCA had they been exclusively presented with monochrome displays. The results of Rubio-Fernández (under review) suggest otherwise, since participants produced zero rates of RCAs when they requested geometrical figures from monochrome displays alone (for a recent investigation of consistency in referential overspecification, see Tarenskeen et al., 2015). Experiment 1 is therefore the first study to specifically investigate the effect of color contrast on the use of RCAs.

The second hypothesis to be investigated in relation to the efficiency of RCAs was whether English speakers would produce more RCAs than Spanish speakers, despite both languages having the same basic color terms. I have recently argued that, in face-to-face referential communication, color adjectives guide an interlocutor’s visual search for the referent (Rubio-Fernández, under review). In this view, color adjectives are a more efficient cue in pre-nominal position than in post-nominal position because in the latter case the hearer’s visual search is initially guided by the noun (and not by the color adjective). It was therefore hypothesized that RCAs would be produced more frequently in English than in Spanish.

The relative efficiency of color adjectives with regards to the incrementality of language processing is best investigated in relatively sparse displays, such as the ones used in Experiment 1. Thus, in a 4-object display, a Spanish hearer may be able to identify the intended referent in processing the noun, thus rendering the post-nominal color adjective useless as a visual cue. The incrementality of language processing is therefore an important factor in the production of RCAs across languages. However, this factor has not been previously investigated either in computational or in pragmatic studies on referential communication.

In order to test the above hypotheses, I designed the Paper Dolls task: a simple referential communication task in which participants had to ask the experimenter to click on paper clothes and shoes in a series of 4-garment displays following a model paper doll. This task also served to test a third hypothesis related to the effect of color pertinence on the production of RCAs: I predicted that both English and Spanish participants in the Paper Dolls task would produce a relatively high proportion of RCAs because color is a central property of clothes and shoes in Western cultures and may therefore feature in reference scripts for such categories. Consider in this respect how color coordination is important when we choose clothes and how some colors are even more fashionable than others, depending on the season. These effects, however, are not observable in all man-made objects, despite the fact that artifacts often come in different colors (e.g., Kitchenware or office supplies). One would therefore expect that the association between color and clothes should be stronger than the association between color and other types of artifacts.

### Method

#### Participants

Thirty-nine undergraduate students from University College London and the University of Kent (UK), all native speakers of English (20 female), and 39 undergraduate students from the Universities of Oviedo and Baleares (Spain), all native speakers of Spanish (25 female), took part in the experiment for monetary compensation. All participants gave consent to have their voice recorded during the experiment. Ethics approval was obtained from University College London and the University of Baleares. Permission to run the experiment was obtained from all departments where data was collected.

#### Materials and Procedure

**FIGURE 1:**
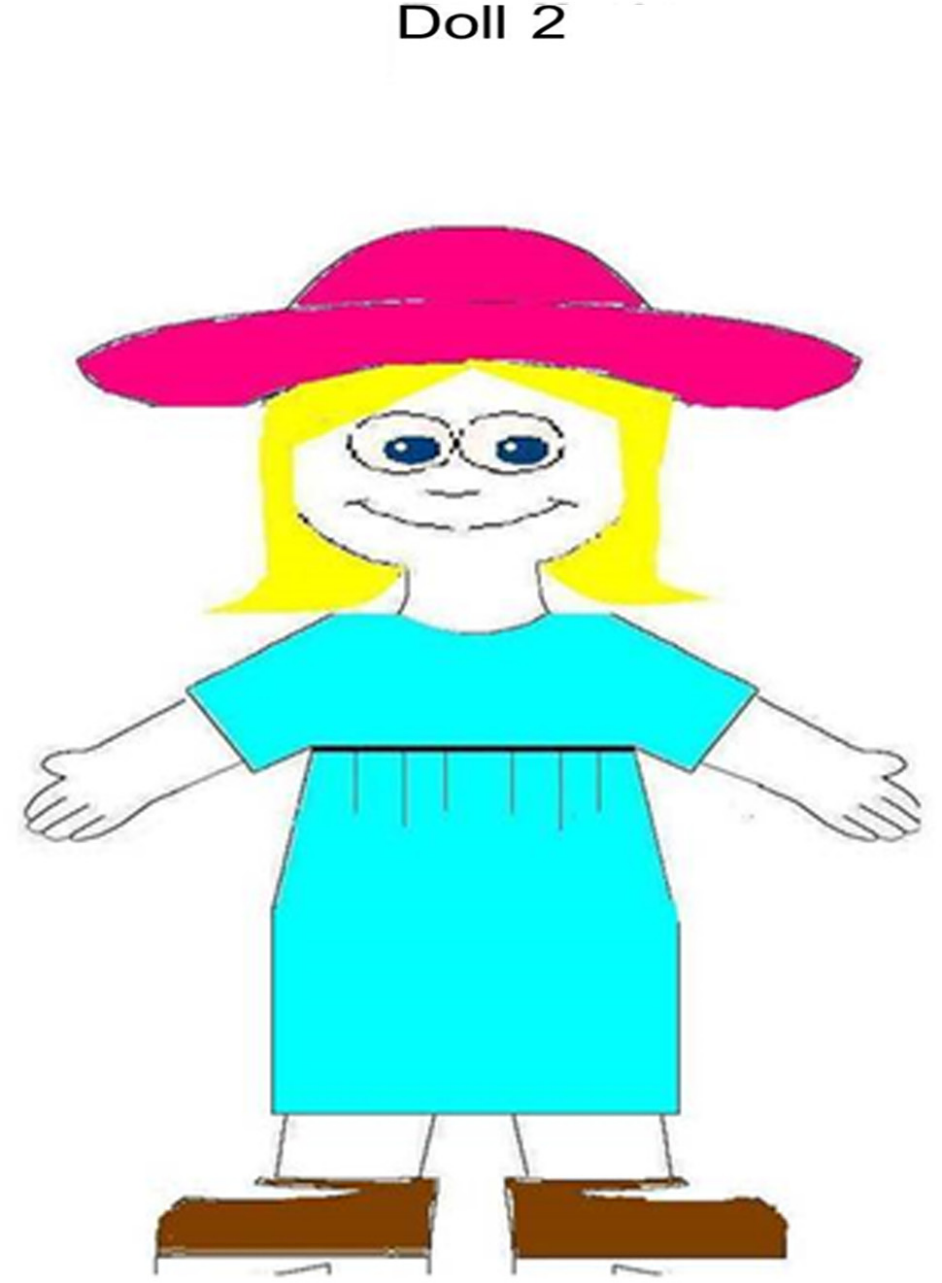
**Model paper doll used in both the monochrome and polychrome conditions**.

**FIGURE 2:**
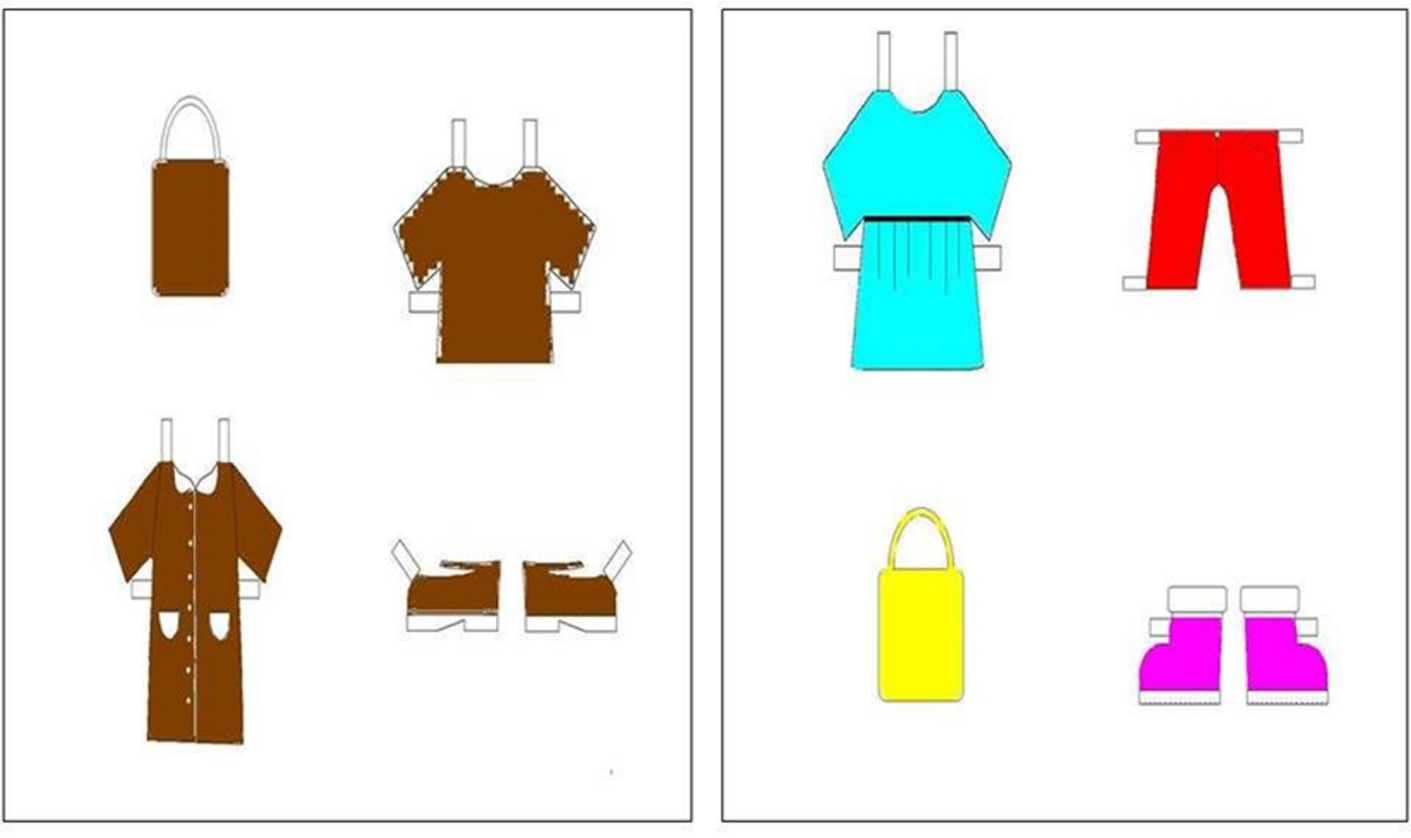
**Displays of paper clothes and shoes from the monochrome condition (left: four different garments of the same color) and the polychrome condition (right: four different garments of four different colors)**.

Six images showing a paper doll were designed so that each doll wore three garments of different colors (see **Figure 1**). Three displays of four paper clothes were constructed for each paper doll, with only one garment corresponding with what the paper doll was wearing (see **Figure 2**). In the polychrome condition, the displays included garments of four different colors. The same displays were used in the monochrome condition, only that all the garments were the same color as the target. Since the model paper dolls always wore 3 garments of different colors, color changed across trials in the monochrome condition (e.g., for the model doll in **Figure 1**, the monochrome displays were pink, blue, and brown). Target garments were the following colors: blue, yellow, green, red, pink, purple, orange, and brown.

Display type (Polychrome vs. Monochrome) and Language (English vs. Spanish) were manipulated between participants in a 2 × 2 design. The paper dolls were printed in color on A4 paper while the 4-garment displays were presented on a computer monitor using E-Prime. Given the simplicity of the task, participants were told that they were a control group in a study investigating the development of children’s communicative skills. The aim of the original study was to see how pre-school children performed in an interactive game in which they had to dress a paper doll following a model and asked the experimenter for the paper clothes they needed. Adults were going to be administered the same task as the children in order to obtain control data to evaluate children’s performance. The only difference with the children’s task was that, instead of playing with cut-out dolls and real paper clothes, adults would be shown paper clothes on a computer monitor and the experimenter would click on their garment of choice in each display.

The experimenter waited until each instruction was completed to click on the designated target (as performing faster may invite Spanish speakers not to encode post-nominal color adjectives). Participants’ requests were recorded and later coded by the experimenter as including or not including a RCA. Only referential expressions including both an adjective and a noun (e.g., ‘The blue dress’ or ‘El vestido azul’) were coded as overspecific.

### Results

The data from both Experiments 1 and 2 were analyzed using non-parametric statistics because they were not normally distributed (which made parametric tests such as ANOVA and *t*-test unsuitable) and because the extreme data values observed in some conditions interfered with model convergence when mixed-model analyses were attempted.

Participants The data from both Experiments 1 and 2 were analyzed using non-parametric statistics because they were not normally distributed (which made parametric tests such as ANOVA and *t*-test unsuitable) and because the extreme data values observed in some conditions interfered with model convergence when mixed-model analyses were attempted. instructions conformed to the minimal or color-overspecific descriptions that were elicited (e.g., ‘The dress’ or ‘The blue dress’). The mean proportions of RCAs for each Language and Display condition in the Paper Dolls task are shown in **Table 1**. A Kruskal–Wallis test was conducted on the proportions of RCAs, revealing a significant difference among conditions, *H*(3) = 41.9, *p* < 0.001.

**Table 1:** Mean proportions of redundant color adjectives (SD) produced in the Paper Dolls task.

Display	Paper Dolls	
	English	Spanish
Polychrome	0.95 (0.15)	0.59 (0.36)
Monochrome	0.37 (0.49)	0.05 (0.17)

Looking first at the effect of Display type, Mann–Whitney tests were carried out on participants’ RCA scores in each language, revealing a significant effect of Display in English, *U* = 71.5, *Z* = 3.32, *p* < 0.001; and in Spanish, *U* = 12.5, *Z* = 4.97, *p* < 0.001, with RCAs being produced more often in polychrome displays than in monochrome displays in the two languages. In addition, of the 40 participants who took part in the polychrome version of the task, 19 participants (17 English speakers) used RCAs systematically, whereas of the 38 participants in the monochrome version, only six participants (all English speakers) used RCAs systematically. A Chi-square test corrected for continuity revealed that the difference between the number of participants who used RCAs systematically (and not systematically) in each type of display is significant, *X*^2^(1,*N* = 78) = 7.60, *p* < 0.006, with more systematic uses of RCAs being observed in the polychrome condition than in the monochrome condition.

Looking at the effect of Language, Mann–Whitney tests were carried out on participants’ RCA scores in each type of display, revealing a significant effect of Language in the Polychrome condition, *U* = 42, *Z* = 4.26, *p* < 0.001; and a marginally significant effect in the Monochrome condition, *U* = 114, *Z* = 1.93, *p* = 0.054, with RCAs being produced more often in English than in Spanish in both types of display. In addition, of the 39 English speakers who took part in the study, 23 used RCAs systematically, while of the 39 Spanish speakers only two used RCAs systematically. A Chi-square test with continuity correction revealed that the difference between the number of participants who used RCAs systematically (and not systematically) in each language is significant, *X*^2^(1,*N* = 78) = 23.6, *p* < 0.001, with English speakers systematically producing RCAs more often than Spanish speakers.

### Discussion

The results of Experiment 1 confirmed that visual-contextual factors affect the production of RCAs: first, RCAs were produced more often in polychrome displays than in monochrome displays, confirming that speakers tend to choose efficient referential expressions when formulating object requests. This pattern of results replicates previous findings by Belke and Meyer (2002) and Koolen et al. (2013), who observed a higher proportion of RCAs in polychrome displays than in monochrome displays. However, a direct comparison with the proportions of RCAs observed in those studies would not be reliable, since they used different types of objects and manipulated a number of other factors (e.g., size contrast and orientation), which may have also affected their results together with the effect of color contrast.

Second, RCAs were produced more often in English than in Spanish, suggesting again that speakers are efficient in their use of RCAs since pre-nominal color adjectives are a more efficient cue to the hearer’s visual search than post-nominal color adjectives (for relevant visual-search studies, see Spivey et al., 2001; Reali et al., 2006; Clarke et al., 2015).

Regarding semantic-category factors, participants in the Paper Dolls task revealed a strong tendency to use RCAs when referring to clothes and shoes, both in the English- and Spanish-Polychrome conditions. In order to evaluate the magnitude of this effect, I will compare the results of Experiment 1 with those reported in Rubio-Fernández (2015). In the latter study, which I conducted in parallel with the present one, I used the Figures and Stickers task: a similar test to the Paper Dolls task in which participants had to ask the experimenter to click on a geometrical figure in a series of 4-figure displays following a model figure. A comparison between these two studies is reliable for two reasons: first, the materials and procedures of the two tasks were identical, with the exception of the shapes used in the displays. Second, color is a central property of clothes and shoes, whereas it is not a particularly central property of geometrical figures. It therefore follows from my prediction regarding color pertinence that English and Spanish speakers should produce more RCAs when performing the Paper Dolls task than when performing the Figures and Stickers task.

The results of Rubio-Fernández (2015) revealed that English speakers produced RCAs 46% of the time in polychrome displays of geometrical figures (*SD*=5.06), while Spanish speakers did so 14% of the time (*SD*=3.03). Relative to the data elicited with geometrical figures, English speakers produced more than twice as many RCAs when referring to clothes and shoes in Experiment 1 (0.46 vs. 0.95), while Spanish speakers did so four times as often (0.14 vs. 0.59). The comparison between the results of the Paper Dolls task and the Figures and Stickers task used by Rubio-Fernández (2015) confirm that people tend to produce RCAs when color is a central property of the noun category, as it is the case with clothes and shoes.

Rubio-Fernández (under review) also tested English participants on the Figures and Stickers task using monochrome displays of geometrical figures. The comparison between the monochrome conditions of the Figures and Stickers task and the Paper Dolls task is critical for the present investigation since the two factors considered in this study (i.e., visual-contextual factors and semantic-category factors) are at odds in those conditions. The question is therefore whether English speakers would produce more RCAs when referring to clothes than to geometrical figures in monochrome displays. If that is the case, semantic-category factors would trump visual-contextual factors since specifying the color of a pair of shoes in a monochrome display, for example, would not facilitate the identification of the shoes for the hearer.

Rubio-Fernández (under review) reported that English speakers produced zero rates of RCAs when referring to geometrical figures in monochrome displays. Relative to these results, the proportion of RCAs observed in the English-Monochrome condition of the Paper Dolls task (0.37) was significantly higher (Mann–Whitney test, *U* = 95, *Z* = 2.48, *p* < 0.014). This pattern of results is revealing, since color does not facilitate object identification when all objects are the same color and therefore, the use of color adjectives to refer to clothes in the monochrome condition was driven by the semantic category of the noun. This effect, however, was only observed in English, with the rates of RCAs produced in the Spanish-Monochrome condition of the Paper Dolls task being close to zero. This is also an interesting difference, since there is no reason to suppose that color is more pertinent for clothes in British than in Spanish culture.

The picture emerging from Experiment 1 is therefore a complex one, with color contrast (monochrome vs. polychrome), adjective position (pre-nominal vs. post-nominal) and semantic category of the noun (clothes and shoes vs. geometrical figures) having a combined effect on the production of RCAs. The interaction of these factors suggests that the production of RCAs is highly context-dependent and requires a finer-grained analysis than a standard evaluation of informativeness. According to such pragmatic analyses, the RCAs observed in the various conditions discussed above would all have been equally over-informative, yet the variability in the data would remain unaccounted for unless other factors were taken into consideration.

## Experiment 2

In addition to the effect of color pertinence investigated in Experiment 1, Experiment 2 investigated a second semantic-category factor; namely the effect of color typicality. More specifically, whether people would use RCAs to refer to objects of atypical colors. Sedivy (2003, 2004) observed that people tend to use RCAs for objects of variable colors (e.g., a blue cup) but not for objects of stereotypical colors (e.g., a yellow banana). Regarding objects atypical colors (e.g., a pink banana), Westerbeek et al. (2015) have recently shown that color tends to be overspecified more often when it is atypical of an object than when it is typical (for a study of shape and material typicality, see also Mitchell et al., 2013). In addition, and relevant to the present argument that RCAs can be efficient in a given visual context, Westerbeek et al. (2015) found that the preference for atypical colors was stronger when color was more important for object identification.

Westerbeek et al. (2015) mainly used displays of fruits and vegetables (although their second experiment also included other stereotypical-color objects) and presented them in more or less typical colors. More in line with the various types of objects used by Sedivy (2003, 2004), Experiment 2 used objects of stereotypical, variable and atypical colors (e.g., an orange carrot, a red bicycle and a pink banana). The aim of Experiment 2 was to investigate the overspecification of atypical colors as a test of the view that RCAs can be efficient, and therefore cooperative in nature (i.e., a test of the pragmatics of color overspecification). For this purpose and unlike the above studies, I manipulated not only color typicality, but also the type of instruction that the participants received at the start of the task (i.e., standard vs. cautionary instructions, with the latter alerting participants to the possibility of a communication breakdown).

There are at least two possible reasons why a speaker may choose to overspecify an atypical color. The first reason would be a bottom-up effect resulting from a violation of the speaker’s word knowledge. For example, a pink banana would be such an odd banana that its color might be highly salient and therefore mentioned in a request for the object even if unnecessary for unique reference. However, there is also a compatible, top-down process by which a speaker would mention the atypical color of an object in order to prevent the hearer from deriving the wrong presupposition. For example, if the speaker wanted a pink banana from among various objects but did not mention its color, the hearer would probably start looking for a yellow object. This hypothesis is supported by the results of a visual-world study by Huettig and Altmann (2011), which showed that when people hear the name of a stereotypical-color entity (e.g., ‘spinach’, which is typically green), they fixate on objects of that color, even though the actual color of the category was not mentioned. Thus, in order to spare the hearer unnecessary effort, the speaker might choose to use a RCA when referring to an atypical-color object.

This second factor would be a pragmatic factor and is related to the question of whether speakers may be cooperative when they use RCAs in their object requests. It is important to note that these two factors are compatible and, in fact, the second, top-down factor depends on the first, bottom-up factor: in order for the speaker to want to spare the hearer unnecessary effort, he must have first detected that the color of the target object violated his world knowledge of the category. Therefore, the aim of Experiment 2 was not to investigate which of these two factors plays a role in the production of RCAs. Instead, the aim of the experiment was to investigate whether speakers may go beyond noticing that the color of a certain object is atypical, and encode RCAs to facilitate object identification for the hearer.

In order to investigate this question, I designed the Yellow Pig task, a simple referential communication task in which participants had to ask the experimenter to click on a target in a series of 4 × 4 displays. In order to investigate the effect of color typicality, the targets were stereotypical-color fruits, vegetables, and animals (e.g., a brown dog); atypical-color fruits, vegetables, and animals (e.g., a pink banana) and variable-color artifacts (e.g., a silver toaster). The latter condition served as a neutral baseline for color typicality, understood as the midpoint between the stereotypical and atypical conditions.

Regarding the question of whether participants are being cooperative when they mention atypical colors, a manipulation was introduced in the instructions intended to make participants more sensitive to a potential communication breakdown between the participant and the experimenter. Participants in the Cautionary condition were made to believe that participants in the pilot phase of the study had sometimes failed to specify which figure was the target and the experimenter had had to ask which of two possible referents they were referring to. Importantly, ambiguity was never an issue in the actual test (with the displays always including different types of figures).

The key hypothesis was that, if participants mentioned atypical colors in order to spare the experimenter unnecessary effort in her object search, a subtle manipulation in the instructions should result in an increase in RCAs in the atypical-color condition but not necessarily in the other two conditions, since only the atypical-color condition would be susceptible to momentary miscommunication. In contrast, if the modified instructions generally increased the salience of color contrast for the participants, then this manipulation should result in an overall increase in the production of RCAs across conditions, and not only in the atypical-color condition.

### Method

#### Participants

Twenty-nine postgraduate students from the University of Groningen (Netherlands) took part in the experiment. Participants were all native speakers of Spanish (15 women) and participated for monetary compensation. The experiment was conducted at the University of Groningen because the author was collaborating in another project at the Psychology Department and the University of Groningen happens to have a large community of Spanish-speaking students. All participants had come to the Netherlands to complete their higher education. Ethics approval was obtained from the Psychology Department in Groningen.

#### Materials and Procedure

**FIGURE 3:**
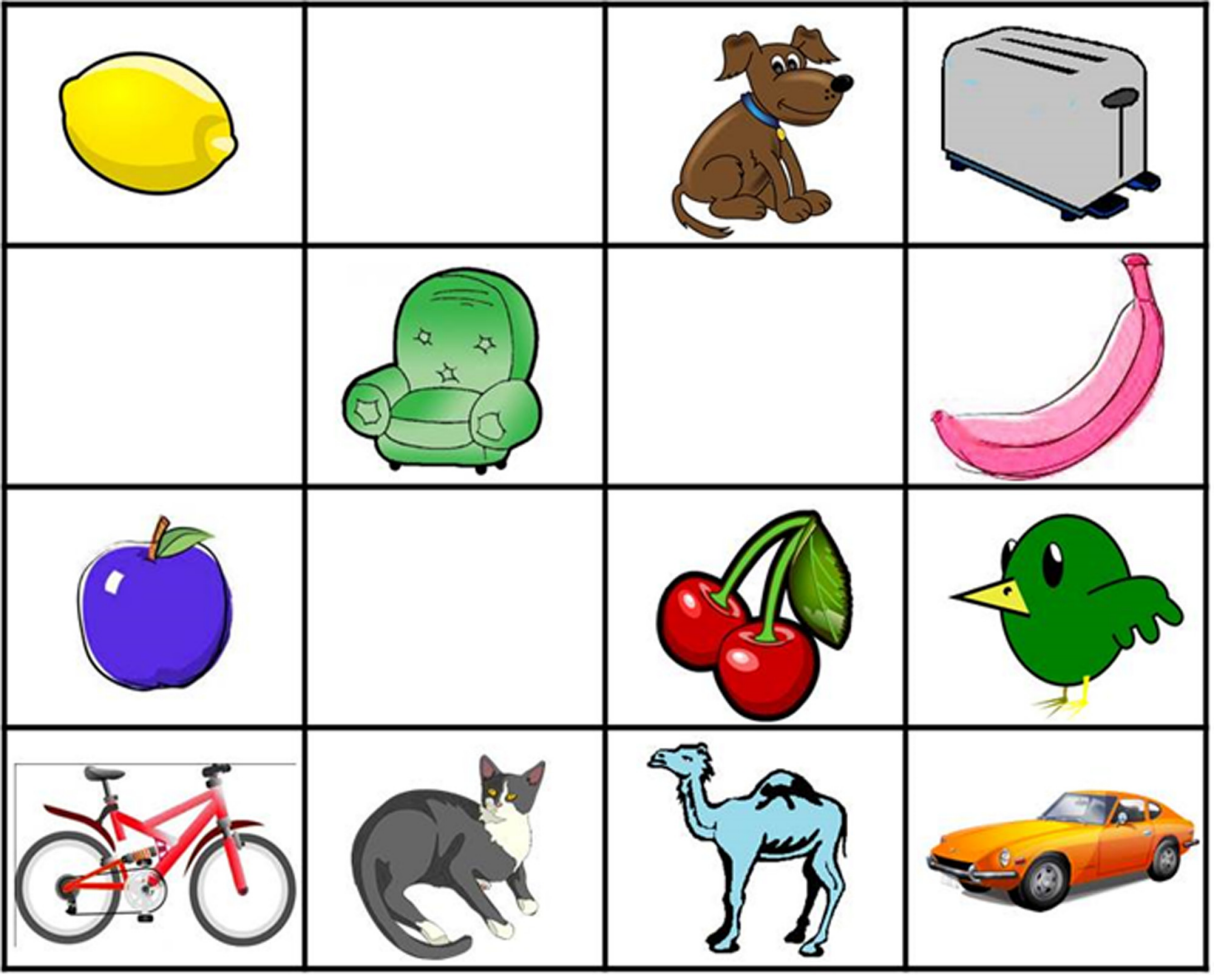
**Grid of objects from the Yellow Pig task.** All grids included two fruits and vegetables of typical colors (yellow lemon and red cherries), two fruits and vegetables of atypical colors (pink banana and purple apple), two animals of typical colors (brown dog and gray cat), two animals of atypical colors (green bird and blue camel) and four artifacts of variable colors (red bike, green sofa, silver toaster, and orange car). In this particular trial the target was the pink banana.

Experiment 2 used 4 × 4 grids in a within-participant design. Sixteen 4 × 4 grids were constructed, each including 12 clip-art objects and 4 empty cells, all randomly distributed in the grid space. Three types of objects were used as targets: animals, fruits and vegetables, and artifacts. These three target types were divided into three categories depending on the typicality of their color: stereotypical, variable and atypical color. Atypical colors never applied to the target category in real life (e.g., a pink banana or a blue camel). Each grid included four animals and four fruits and vegetables, two of each in stereotypical colors and two in atypical colors, plus four artifacts in variable colors. During the experiment participants had to refer to five items of each color type in a fixed random order. All the objects in the grids were different and so the use of color adjectives was redundant in all trials (see **Figure 3**). The first trial was treated as a warm-up and was not analyzed.

The grids of objects were presented on a computer monitor using E-Prime. Participants had to ask the experimenter to click on a specific object in each grid. For the participants’ materials, fifteen 4 × 4 blank grids were printed on paper. A cross was placed in each blank grid in the cell that corresponded with the target object in the computer display. In order to facilitate synchronization between the experimenter and the participant, the grids of objects and the blank grids were numbered.

Participants had to ask the experimenter to click on the object in the display that corresponded with the cross on their blank grid. Participants sat behind the experimenter so that the experimenter could not see their paper grids but they could see the computer monitor in front of the experimenter. Participants were (falsely) told that the computer program randomized the objects in the grids and the experimenter was therefore naïve as to which object participants would ask for in each trial.

Given the simplicity of the task, participants were told that they were a control group in a developmental study investigating children’s abilities to navigate two-dimensional spaces. Two types of instructions were used, Standard and Cautionary. In both instructions participants were told that they were going to play an interactive game in which they had to tell the experimenter to click on a specific figure in a grid of objects on the computer screen, using a cross on an empty paper grid to identify the target. The Cautionary instructions were identical to the Standard instructions with the exception of a paragraph at the end of the text in which participants were (falsely) told that in the pilot phase of the study, communication had sometimes broken down because participants did not pay enough attention to the objects in the grid and failed to notice that there were two objects of the same category. A false example was given in which a participant asked the experimenter to ‘click on the box’ and the experimenter had to ask which one: the big box or the small box. In reality, the grids of objects never included two objects of the same category, and so ambiguity was not an issue in any of the trials. Also, color was not mentioned in either type of instructions to avoid making it salient.

The whole task was administered in Spanish. The results of Experiment 1 suggest that English participants may have produced more RCAs than Spanish speakers. This, however, does not affect the predictions tested in Experiment 2 since the same pattern of results would be predicted for both groups of speakers across conditions (although the proportions of RCAs observed in each condition may have been higher for English speakers). Participants’ requests were recorded and later coded by the experimenter as including or not including a RCA. Only referential expressions including both an adjective and a noun (e.g., ‘El plátano rosa’ – the pink banana) were coded as overspecific.

### Results

Participants instructions conformed to the minimal or color-overspecific descriptions that were elicited (e.g., ‘El plátano’ or ‘El plátano rosa’ – the banana or the pink banana). The mean proportions of RCAs for each Color and Instruction type are shown in **Table 2**. Looking first at the effect of color typicality on the production of RCAs, a Friedman test revealed a significant difference among the three Color types across the two Instruction types, *X*^2^(2) = 28.2, *p* < 0.001. *Post hoc* analyses of participants’ RCA scores with the Wilcoxon test revealed that RCAs were produced significantly more often in the Atypical-color condition than in the Stereotypical-color condition, *Z* = -3.47, *p* < 0.002; also significantly more often in the Variable-color condition than in the Stereotypical-color condition, *Z* = -2.89, *p* < 0.005; and significantly more often in the Atypical-color condition than in the Variable-color condition, *Z* = -3.45, *p* < 0.002.

**Table 2:** Mean proportions of redundant color adjectives (SD) produced in each color and instruction condition of the Yellow Pig task.

Instructions	Color		
	Stereotypical	Variable	Atypical
Standard	0 (0)	0.06 (0.15)	0.14 (0.36)
Cautionary	0 (0)	0.16 (0.17)	0.67 (0.38)

*The task was conducted with Spanish speakers.*

Looking at the effect of Instruction type, as expected, no effect was observed on the production of RCAs in the Stereotypical-color condition, in which color terms were never used. The effect of Instruction type was non-significant in the Variable-color condition, *U* = 68, *Z* = -1.92, *p* = 0.112. In contrast, participants produced RCAs in the Atypical-color condition significantly more often after having received Cautionary instructions than after having received Standard instructions, *U* = 36, *Z* = -3.24, *p* < 0.003.

In addition, of the 14 participants in the Standard-instructions condition, 12 did not produce RCAs in any of the trials. Of the 15 participants in the Cautionary-instructions condition, only two did not use RCAs in any of the trials. A Chi-square test with continuity correction revealed that the difference between the number of participants who never produced RCAs (and those who sometimes did) in the two Instruction conditions is significant, *X*^2^(1,*N* = 29) = 12.4, *p* < 0.001, with more participants never producing RCAs in the Standard-instruction condition than in the Cautionary-instructions condition.

Looking at item effects, the 10 participants who produced RCAs in the Variable-color condition (following either type of Instructions) always did so in the same one or two consecutive trials (i.e., a red pencil and/or a green cup). Moreover, 6 of these 10 instances were potential carry-over effects from a previous Atypical trial in which participants had overspecified color. This pattern of results suggests that the increase in the use of RCAs observed in the Variable-color condition between the Standard and the Cautionary instructions did not generalize over items (and was potentially related to the effect observed in the Atypical-color condition). By contrast, participants’ use of RCAs in the Atypical-color condition was observed across all items in that category, thus revealing a more reliable effect of Instruction type.

#### Post-test

In order to rule out the possibility that the results of Experiment 2 reflected differences in the relative saliency of the target color in the different displays, grayscale saliency maps were created for the 15 slides employed in the study using Achanta et al. (2009)’s algorithm. The saliency maps were given to two naïve coders who ranked the 12 objects in each display according to their perceived salience (with white objects and black objects corresponding with the most and least salient objects in the display, respectively). Only the ranking of the target object was computed, with the highest ranking being adopted by default when there was a disagreement. The average ranking of the targets in the Variable-color condition was 6.6 (range: 8, 2, 5, 6, 12), in the Atypical-color condition was 5.6 (range: 2, 11, 2, 7, 6) and in the Stereotypical-color condition was 6.6 (range: 10, 4, 3, 12, 4). The results of this post-test using grayscale saliency maps suggest that the tendency to overspecify the color of atypical-color objects was not triggered by these targets being more perceptually salient than the targets in the Variable-color and Stereotypical-color conditions.

### Discussion

The results of Experiment 2 confirm that color typicality has an effect on the production of RCAs in objects requests: while stereotypical colors were never used redundantly, variable colors were used redundantly significantly more often. This pattern of results replicates, with Spanish speakers, those reported by Sedivy (2003, 2004) with English speakers. As predicted, atypical colors were used redundantly significantly more often than variable and stereotypical colors. The difference between atypical and variable colors is particularly revealing, since the Variable-color condition was a neutral baseline for color typicality (i.e., the color of variable-color artifacts was neither stereotypical nor atypical). In line with the results reported by Westerbeek et al. (2015) with Dutch speakers, the results of Experiment 2 suggest that the less typical a color is for a given category token, the more likely it will be encoded in a request for the object.

The pattern of results observed in the Stereotypical and Atypical conditions with standard instructions is comparable to the results of Westerbeek et al. (2015). However, the proportion of RCAs for atypical color targets was much higher in Westerbeek et al. (2015) than in Experiment 2 (approximately 0.75 vs. 0.14, respectively). Leaving aside potentially important differences in the actual materials that were used in the two studies (which differed in type of objects and colors), a possible explanation for this difference is that Dutch speakers encode adjectives pre-nominally, while Spanish speaker do so post-nominally. The different results observed with these two groups of speakers therefore parallels the difference observed in Experiment 1 between English and Spanish speakers, indirectly supporting the hypothesis that adjective position is an important factor in the production of RCAs in face-to-face referential communication.

Regarding the issue of whether speakers are being cooperative when they mention atypical colors in object requests, the results of Experiment 2 suggest that participants may have tried to prevent the hearer from deriving the wrong presupposition and looking for a stereotypical target. Thus, those participants who received the cautionary instructions did not adopt a general strategy to describe the color of all types of targets in order to aid communication; instead, they did so mostly when referring to atypical-color objects, which were the only targets that could have caused momentary miscommunication. I interpret these results as evidence that RCAs can be cooperative in nature, although other factors and considerations may also be at play (e.g., the position of the adjective or the pertinence of color for the noun category, as suggested by the results of Experiment 1).

## General Discussion

Contrary to standard pragmatic models in the Gricean tradition, I have argued that speakers may be efficient when they produce RCAs in referential communication. If this is the case, speakers should produce RCAs in those situations in which they could reasonably expect that color would facilitate the hearer’s visual search for the referent. An efficiency-based analysis of color overspecification is finer-grained than standard pragmatic analyses in terms of informativeness, and can therefore explain a number of perceptual factors that should affect a speaker’s choice of referential expression (provided the speaker is rational and cooperative, as Gricean models assume).

As predicted, the production of RCAs in the present study was affected both by visual-contextual and semantic-category factors. Thus, speakers produced significantly more RCAs in polychrome displays than in monochrome displays, and did so more often when the adjective appeared in pre-nominal position (English) than in post-nominal position (Spanish). The results of Experiment 1 therefore suggest that speakers tend to produce RCAs when color may facilitate object identification for the hearer, hence behaving efficiently. This conclusion was also supported by the results of Experiment 2, where participants produced more RCAs when they were alerted to possible communication difficulties, suggesting that the use of RCAs can be cooperative in nature.

Semantic-category factors related to world knowledge also affected the production of RCAs, with English speakers producing twice as many RCAs when referring to clothes than to geometrical figures, and Spanish speakers producing four times as many (Rubio-Fernández, 2015). Moreover, English speakers produced significantly more RCAs when referring to clothes than when referring to geometrical figures in monochrome displays (Rubio-Fernández, under review). Finally, participants in Experiment 2 produced more RCAs for entities of atypical colors than for entities of variable and stereotypical colors, suggesting that our world knowledge affects our use of color adjectives; for example, when the color of an object violates our expectations (and hence those of our interlocutors).

The results of this study have implications for computational models of reference production, in particular Dale and Reiter’s (1995) classic Incremental Algorithm, which incorporates Pechmann’s (1989) finding that salient attributes such as color are sometimes overspecified. Because the Incremental Algorithm selects attributes in a preferred order, it is computationally simple and easy to implement (for a review of this and related algorithms, see Krahmer and van Deemter, 2012). However, from a psycholinguistic point of view, the Incremental Algorithm fails to incorporate the multiple factors that may affect the production of RCAs in referential communication. For example, the results of the present study show that in an otherwise identical situation, the use of color adjectives may vary depending on the syntactic position of the adjective (pre-nominal vs. post-nominal), the semantic category of the referent (e.g., comparable displays of clothes vs. geometrical figures), the typicality of the color of the referent (e.g., a yellow banana vs. a pink banana), and the speaker’s disposition to maximize the chances of successful communication (see also Koolen et al., 2011).

Insensitive to all these sources of variation, the Incremental Algorithm produces RCAs because it treats color as a preferred attribute and never withdraws attributes once they have been selected (not even when the later inclusion of another attribute would render color redundant). However, because the algorithm checks the category of the object before its color, it never overspecifies color if the category is unique in the context (contrary to what was observed in this and other studies). Also, the Incremental Algorithm only overspecifies color if it has discriminatory value (e.g., it would never generate a color adjective in a monochrome display, contrary to what was observed in the Paper Dolls task). The results of this study therefore call for a more nuanced treatment of color in computational models of reference production, besides making it a preferred attribute that may be overspecified in very specific situations (for a discussion of various probabilistic revisions to the Incremental Algorithm, see Krahmer and van Deemter, 2012; van Deemter et al., 2012).

The results of this study also have implications for pragmatic models of reference production, which so far have failed to take perceptual factors into consideration. More specifically, I want to challenge the view that the use of RCAs is ‘non-contrastive,’ as opposed to those uses that are intended to establish a contrast between two objects of the same kind (Sedivy, 2004, 2007; Grodner and Sedivy, 2011). In my view, participants in this and earlier studies may have used RCAs in order to exploit a color contrast among different types of objects (e.g., Belke and Meyer, 2002; Sedivy, 2003; Koolen et al., 2013). Thus, participants may have asked the experimenter for ‘the blue cup,’ for example, in a situation where there was only one cup; however, if the cup was the only blue object in the display, then color would have been used contrastively. This interpretation of the effect of color contrast on the production of color adjectives calls for a revision of the pragmatic notion of *referential contrast*.

The canonical function of an adjective in an object request is to exploit a contrast between the intended referent and other objects of the same kind, which would allow the interlocutor to uniquely identify the target object against its competitors (e.g., a plastic cup vs. a paper cup). Contrary to the standard view, I want to propose that in the case of prenominal color adjectives, referential contrast may be established across categories, rather than within a given category (the way it is established for material and relative adjectives; Sedivy, 2003, 2004). According to this definition, prenominal color adjectives are used contrastively whenever there is a color contrast in the visual context that the speaker could exploit for efficient referential communication (e.g., a blue cup vs. a red jug). Those situations where prenominal color adjectives are used contrastively in the canonical sense of the term to distinguish between two objects of the same kind (e.g., a blue cup vs. a red cup) are merely a special case of color-contrastive uses.

## Does Color Overspecification Pose a Challenge to Gricean Pragmatics?

According to Grice’s (1975) Maxim of Quantity, speakers should try to provide their interlocutors with as much information as they need, but not more. The extent to which overspecification poses a challenge to Gricean models of referential communication has been a recurrent theme in the pragmatics literature (e.g., Sedivy, 2003, 2004, 2007; Engelhardt et al., 2006; Grodner and Sedivy, 2011; Heller et al., 2012). The most extreme position in this debate has been adopted by Engelhardt et al. (2011), who went so far as to argue that overspecific referential expressions ‘impair comprehension’ (but cf. Sonnenschein and Whitehurst, 1982; Mangold and Pobel, 1988; Maes et al., 2004; Davies and Katsos, 2010; Arts et al., 2011b). The results of the study by Engelhardt et al. (2011) are not entirely surprising, however, since these authors investigated the effect of size and color overspecification using minimal displays of two figures in which the hearer had to identify a target following a modified description (e.g., ‘the red square’ or ‘the big star’). Given the simplicity of the hearer’s visual search, it is only to be expected that color would not facilitate object identification when the display included two different figures (e.g., a red square and a blue circle vs. a red square and a blue square).

Two patterns of results seem to support this interpretation of the results of Engelhardt et al. (2011): eye-tracking studies of adjectival modification (e.g., Sedivy et al., 1999; Sedivy, 2003, 2004; Rubio-Fernández, under review) have repeatedly shown that listeners are able to visually identify a referent as soon as they have enough information to do so, even when the adjective is redundant. In Engelhardt et al.’s (2011) study, size and color were distinctive properties of the target in both the redundant and the contrastive conditions, which means participants should have been able to visually identify the target referent in hearing the adjective. However, Engelhardt et al. (2011) did not use eye tracking to measure reference resolution during processing; instead, they asked participants to press a right/left key to indicate the position of the target on the screen. The longer response times observed in the redundant condition suggest that participants’ responses did not measure visual identification alone (which should have been comparable in both conditions), but also reflected an implicit pragmatic judgment by comparison to the contrastive condition.

One reason why participants may have found the overspecific descriptions in Engelhardt et al. (2011) pragmatically infelicitous is because they were unnatural in the visual context: Rubio-Fernández (under review) observed that speakers never overspecified the size of a target in a 2-figure display, and did so less than 25% of the time with color adjectives. When using larger displays, however, both size and color were overspecified over 60% of the time. That the overspecified descriptions used by Engelhardt et al. (2011) were highly unnatural might also explain the early N400 observed in that condition, which the authors interpreted as reflecting either a semantic integration problem or low predictability.

On reflection, what is more remarkable in the study by Engelhardt et al. (2011) is their interpretation of their results as in line with Grice’s (1975) model of verbal communication. After all, Grice’s model rests not only on the Maxim of Quantity, but most importantly on the Cooperative Principle and the general assumption that speakers and hearers interact as rational agents. Therefore, if redundant referential expressions impair communication, as Engelhardt et al. (2011) claim, why do speakers overspecify their referential expressions as often as they do? One would assume that a rational and cooperative speaker who had a choice between referring to ‘the t-shirt’ or ‘the yellow t-shirt’ would not choose (systematically, sometimes) the modified description if that would impair the hearer’s comprehension.

It seems safe to assume that speakers are being rational and cooperative when they produce RCAs that could facilitate the interlocutor’s search for the referent (e.g., ask for ‘the blue cup’ in a situation where there is only one cup, but it is also the only blue object in a relatively dense display). But what about those RCAs that are produced in monochrome displays? In the present study only English speakers produced such RCAs when referring to clothes, while Spanish speakers did not. Moreover, Rubio-Fernández (under review) reports that English speakers produced zero rates of color overspecification in monochrome displays of geometrical figures using the same task. These results suggest that the tendency to use RCAs in monochrome displays observed in Experiment 1 was driven by the pertinence of color for clothes and the general tendency of English speakers to overspecify color.

However, using RCAs to refer to clothes cannot be considered as irrational or un-cooperative behavior since the pertinence of color for clothes may be so high that hearers expect that the color of clothes be encoded in referential communication. Along these lines, Dale and Reiter (1995) have argued that one reason why speakers may sometimes use RCAs when color has no discriminatory power in the context is because they are using reference scripts that determine which attributes are expected for a certain semantic category (for a related view using ‘default descriptions,’ see Sedivy, 2003, 2004, 2007; Grodner and Sedivy, 2011). This could be the case for the color of clothes and shoes in the English language, as suggested by collocations such as ‘black tie’, ‘little black dress,’ ‘the red shoes’ or ‘white collar workers.’

## Conclusion

Those color adjectives that are not necessary to establish unique reference are traditionally considered redundant or over-informative, even though they may be efficient (insofar as they may facilitate the interlocutor’s search for the object) and/or pertinent for the requested object (insofar as color is important for the semantic category). Therefore, traditional pragmatic analyses cast in terms of informativeness alone fall short of explaining the ubiquitous use of RCAs in referential communication and the kind of factors that affect these uses. An analysis in terms of efficiency and pertinence, however, reveals that the use of RCAs is in line with Gricean pragmatics.

## Author Contributions

The author confirms being the sole contributor of this work and approved it for publication.

## Conflict of Interest Statement

The author declares that the research was conducted in the absence of any commercial or financial relationships that could be construed as a potential conflict of interest. The reviewer RK and handling Editor declared their shared affiliation, and the handling Editor states that the process nevertheless met the standards of a fair and objective review.
